# Prevalence and Risk Factors of Latent Tuberculosis Infection Detected by IGRA in Patients with Immune-Mediated Inflammatory Diseases Before and During Biologic DMARD Therapy (TITAN Study)

**DOI:** 10.3390/jcm14144990

**Published:** 2025-07-15

**Authors:** José Antonio Mata-Marín, Marisol Apaez-Iglesias, Ana Luz Cano-Díaz, Juan Pablo Sánchez-Navarro, Diana Edith Fernández-Madinaveitia, Gustavo Barriga-Angulo, Salma Triana-González, Alberto Chaparro-Sánchez, Ericka Nelly Pompa-Mera, Jesús Enrique Gaytán-Martínez

**Affiliations:** 1 Infectious Diseases Department, Hospital de Infectología “Dr Daniel Méndez Hernández”, Centro Médico Nacional “La Raza”, Instituto Mexicano del Seguro Social, Mexico City 02990, Mexico; jamatamarin@gmail.com (J.A.M.-M.); marisol.apaez.iglesias@gmail.com (M.A.-I.); jp_sanchezn@hotmail.com (J.P.S.-N.); stg.9204@gmail.com (S.T.-G.); a_chaparro@hotmail.com (A.C.-S.); jgaytanmtz@yahoo.com.mx (J.E.G.-M.); 2 Dermatology Department, Hospital General de Zona 1 A “Dr Rodolfo Antonio de Mucha Macías”, Instituto Mexicano del Seguro Social, Mexico City 03300, Mexico; madinaveitia79@hotmail.com; 3 Laboratory Department, Hospital de Infectología “Dr Daniel Méndez Hernández”, Centro Médico Nacional “La Raza”, Instituto Mexicano del Seguro Social, Mexico City 02990, Mexico; gustavo.barriga@imss.gob.mx; 4 Research Unit, Hospital de Infectología “Dr Daniel Méndez Hernández”, Centro Médico Nacional “La Raza”, Instituto Mexicano del Seguro Social, Mexico City 02990, Mexico; erickanelly@yahoo.com.mx

**Keywords:** latent tuberculosis infection, immune-mediated inflammatory diseases, biologic DMARDs, QuantiFERON-TB gold plus, Mexico

## Abstract

**Background/Objectives**: Patients with immune-mediated inflammatory diseases (IMIDs) treated with disease-modifying antirheumatic drugs (DMARDs) are at increased risk of latent tuberculosis infection (LTBI) reactivation, influenced by DMARD type. This study aimed to determine LTBI prevalence using interferon-gamma release assays (IGRAs) and identify associated risk factors in IMID patients in a middle-high TB burden setting in Mexico. **Methods**: A cross-sectional study was conducted from July 2024 to April 2025 at an IMID clinic. Patients aged ≥18 years, either receiving DMARDs or prior to initiating treatment, were included. LTBI was diagnosed using the QuantiFERON-TB Gold Plus assay. Bivariate analysis was performed using the chi-square test, and multivariate analysis was conducted. **Results**: LTBI prevalence was 34.2% (95% CI 29.1–39.7%) according to QFT-Plus and 35.6% (95% CI 29.7–42.0%) according to TSTs (n = 230). Prior TB exposure was the strongest risk factor (aOR 4.20, 95% CI 1.74–10.12, *p* = 0.001), while rheumatoid arthritis was associated with a lower LTBI likelihood (aOR 0.31, 95% CI 0.16–0.59, *p* < 0.001). **Conclusions**: A high prevalence of LTBI was observed in patients with IMIDs treated with DMARDs. Prior tuberculosis exposure was strongly associated with LTBI. These findings highlight the importance of LTBI screening in this population to prevent reactivation.

## 1. Introduction

Tuberculosis (TB) remains a critical global health issue, ranking among the top ten causes of mortality worldwide and the leading infectious causes of death in low- and middle-income countries [1]. In 2023, the World Health Organization (WHO) reported 10.6 million new TB cases and 1.3 million deaths globally, with Latin America and other developing regions disproportionately affected [2]. Latent tuberculosis infection (LTBI), defined by a persistent immune response to *Mycobacterium tuberculosis* without active disease, affects roughly one-quarter of the global population and poses a significant risk for reactivation in high-risk groups [3].

This risk is particularly elevated in patients with immune-mediated inflammatory diseases (IMIDs), such as rheumatoid arthritis, psoriasis, and inflammatory bowel disease, due to immunosuppression from both the disease and its treatments, notably biologic disease-modifying antirheumatic drugs (DMARDs) like tumor necrosis factor-alpha (TNF-α) inhibitors [4,5]. The risk of LTBI reactivation varies by drug class, with TNF-α inhibitors conferring a higher risk compared to non-TNF biologics, such as anti-IL-17 or anti-IL-23 agents [4,6].

The WHO recommends LTBI screening using the tuberculin skin test (TST) or interferon-gamma release assays (IGRAs), such as QuantiFERON-TB Gold Plus, to guide preventive therapy in high-risk populations, including those with IMIDs [7]. The TST is widely used but limited by false positives in individuals vaccinated with Bacillus Calmette-Guérin (BCG) or exposed to non-tuberculous mycobacteria, a common issue in Latin America, where BCG vaccination is nearly universal [8,9]. The IGRA, with its superior specificity and sensitivity, is preferred for immunocompromised populations, yet its use is limited in resource-constrained settings [9,10]. Global LTBI prevalence in IMID patients varies widely. Studies using TST have reported 29.5% in Brazil, 29% in Peru, and 20–30% in other Latin American countries like Colombia and Argentina, while IGRA-based estimates are generally lower due to reduced false positives [11,12,13,14]. In Latin America, high-TB-burden countries like Brazil, Peru, and Mexico (with a TB incidence of 23 cases per 100,000 inhabitants in 2021) face unique challenges, including regional disparities in TB incidence, urban overcrowding, and socioeconomic factors that amplify transmission [15,16,17].

In Latin America, TB epidemiology is heterogeneous, with countries like Mexico, Brazil, and Peru reporting higher burdens in urban and underserved areas, such as Mexico City, São Paulo, and Lima [15,18]. The widespread use of BCG vaccination across the region complicates LTBI diagnosis, as TST results are often confounded [19,20]. The rising prevalence of IMIDs and increasing use of biologic DMARDs in Latin America, driven by improved access to specialized care, heighten the need for robust LTBI screening protocols to prevent reactivation [21]. However, limited access to IGRA testing and the reliance on TST in many Latin American countries hinder accurate prevalence estimates, particularly for diverse IMID populations with diseases other than rheumatoid arthritis [16,22]. This study aimed to determine the prevalence of LTBI using the IGRA and identify associated risk factors in patients with IMIDs, either before initiating or during treatment with biologic DMARDs, in a high-TB-burden Latin American setting. By addressing regional data gaps and leveraging the IGRA’s diagnostic precision, this study aimed to determine LTBI prevalence using interferon-gamma release assays (IGRAs) and identify associated risk factors in IMID patients in a middle-high TB burden setting in Mexico.

## 2. Materials and Methods

### 2.1. Subjects

Patients aged ≥18 years diagnosed with immune-mediated inflammatory diseases (IMIDs), such as rheumatoid arthritis, psoriatic arthritis, ankylosing spondylitis, systemic lupus erythematosus, and inflammatory bowel disease, were enrolled. Patients were referred to the infectious diseases clinic at the Hospital de Infectología, National Medical Center “La Raza,” Mexico City, for tuberculosis (TB) risk assessment either prior to initiating biologic disease-modifying antirheumatic drugs (DMARDs) or during ongoing DMARD treatment. Only patients with no prior history of active TB or latent TB infection (LTBI) were included.

Patients were excluded if they lost social security coverage before obtaining all laboratory tests, had a history of active TB or previously treated LTBI, or were unable to provide informed consent due to cognitive impairment or other reasons.

### 2.2. Research Design

A cross-sectional study was conducted from 1 July 2024 to 30 April 2025 at the Hospital de Infectología, National Medical Center “La Raza,” a tertiary care and national reference center for infectious diseases in Mexico City, Mexico. This facility serves a diverse population, including patients from high- and low-TB-burden regions across Mexico.

### 2.3. Ethical Considerations

The study protocol was approved by the Ethical and Research Committee of the National Medical Center “La Raza” (Approval No. R-2024-3502-133). Written informed consent was obtained from all participants prior to enrollment, after detailing the study’s purpose, procedures, risks, benefits, and confidentiality measures. The study adhered to the Declaration of Helsinki and local ethical guidelines.

### 2.4. Data Collection

Clinical data were collected through structured interviews, medical record reviews, and laboratory testing during routine clinic visits. The following clinical variables were recorded:

IMID Diagnosis: Specific IMID type (e.g., rheumatoid arthritis, psoriatic arthritis) confirmed by a dermatologist, rheumatologist, or gastroenterologist based on standard diagnostic criteria.

Medication History: Current and prior use of biologic DMARDs (e.g., TNF-α inhibitors, IL-17 inhibitors), synthetic DMARDs (e.g., methotrexate, leflunomide), and Janus kinase inhibitors (JAKi); the duration of DMARD therapy (in months); and specific drug names.

Comorbidities: Presence of hypertension, type 2 diabetes mellitus, chronic kidney disease, or other chronic conditions, verified through medical records and by direct interview.

Serological Testing: Results for hepatitis B virus (HBV; HBsAg, anti-HBc, anti-HBs), hepatitis C virus (HCV; anti-HCV), and human immunodeficiency virus (HIV; anti-HIV), performed using standardized commercial assays.

Lifestyle Factors: Self-reported tobacco use (current, former, never; pack-years for smokers) and alcohol consumption (frequency and quantity).

Sociodemographic Data: A standardized questionnaire was administered to collect information regarding occupation, educational level, BCG vaccination, geographic data, and TB exposure.

LTBI Diagnosis: LTBI was diagnosed using the QFT-Plus (Qiagen, Hilden, Germany) assay, performed according to manufacturer instructions. A positive result was defined as a TB antigen (TB1 or TB2) minus nil value of ≥0.35 IU/mL and at least 25% of the nil value, in accordance with QFT-Plus guidelines. When available, tuberculin skin test (TST) results were recorded, with an induration ≥ 5 mm considered positive in this immunocompromised population. Blood samples for QFT-Plus were collected by trained phlebotomists, processed within 8 h, and analyzed in a certified laboratory. The TST was performed using 0.1 mL (5 TU) of purified protein derivative (PPD), with results read 48–72 h later by trained personnel.

Data Management: Data were recorded in a secure electronic database with access restricted to authorized study personnel.

### 2.5. Statistical Analysis

#### 2.5.1. Sample Size Calculation

The sample size was calculated to estimate LTBI prevalence with a 95% confidence level and a 5% margin of error, assuming an expected prevalence of 14% based on prior studies of rheumatoid arthritis cohorts in Mexico [11]. Using the formula for prevalence studies, where (Z = 1.96), (*p* = 0.14), and (E = 0.05), a minimum of 185 patients was required. To account for potential dropouts (20%), a target of 222 patients was set.

Descriptive Statistics: LTBI prevalence was calculated as the proportion of QFT-Plus-positive patients, with 95% confidence intervals (CIs). Categorical variables (e.g., IMID type, comorbidities, TB exposure) were reported as frequencies and percentages. Continuous variables (e.g., age, duration of DMARD therapy) were assessed for normality using the Kolmogorov–Smirnov test. Normally distributed variables were expressed as means with standard deviations (SDs), while non-normally distributed variables were reported as medians with interquartile ranges (IQRs).

Bivariate Analysis: Associations between LTBI (QFT-Plus positivity) and potential risk factors (e.g., age, sex, IMID type, DMARD use, TB exposure, BCG vaccination) were evaluated using chi-square tests for categorical variables with expected cell counts ≥5, or Fisher’s exact tests for smaller cell counts. Continuous variables were compared using Student’s *t*-test (normal distribution) or the Mann–Whitney U test (non-normal distribution).

#### 2.5.2. Multivariate Analysis

Variables with *p* ≤ 0.25 in bivariate analysis were included in a multivariate logistic regression model to identify independent risk factors for LTBI. Adjusted odds ratios (aORs) with 95% CIs were calculated. Backward stepwise selection was used to refine the model, retaining variables with *p* ≤ 0.05.

Software and Significance: All analyses were performed using IBM SPSS Statistics version 30 (IBM Corp., Armonk, NY, USA) on macOS Ventura 13.6.4. Statistical significance was defined as *p* ≤ 0.05 (two-tailed). Missing data were handled using listwise deletion for multivariate analysis, with sensitivity analyses conducted to assess the impact of missing data on results.

## 3. Results

### 3.1. Patient Characteristics

Of the 329 patients screened, 25 (7.6%) were excluded due to a history of TB or previously diagnosed LTBI, resulting in a final cohort of 304 patients. The cohort was evenly distributed by sex; 154 (50.7%) were men, with a median age of 53 years [IQR 39–61]. Most patients, 211 (69.4%), had a middle–high or professional educational level. Predominant occupations included merchants—83 (27.6%), professionals—60 (19.7%), and domestic service workers—35 (11.5%). Geographically, 274 (90.1%) resided in central Mexico, primarily Mexico City—195 (64.4%) and Mexico State—53 (17.4%); 22 (7.2%) were from high-TB-incidence states (e.g., Veracruz, Guerrero). Two patients were naturalized Mexicans from Bolivia and Venezuela. Common comorbidities included systemic arterial hypertension, in 94 (30.9%); diabetes, in 68 (22.4%); and metabolic syndrome, in 25 (8.2%). Tobacco use was reported by 104 (34.5%), and alcohol consumption in 96 (31.6%). HIV was found to be positive in 3 (1.0%), HCV in 1 (0.3%), and HBV in 1 (0.3%). These infections were infrequent (Table 1).

### 3.2. IMID and DMARD Characteristics

IMIDs were predominantly dermatological, appearing in 186 (61.1%), with psoriasis, in 126 (41.4%), being the most common, followed by rheumatoid arthritis, in 47 (15.4%); ankylosing spondylitis, in 31 (10.1%); and hidradenitis suppurativa, in 31 (10.1%) (Table 2). Anti-TNF-α agents were prescribed to 180 (59.5%) of patients, with adalimumab, in 132 patients (43.4%), followed by anti-IL-17 secukinumab, in 47 (15.4%), being the most frequent. Prior DMARD use was reported in 137 (45%), with anti-TNF-α agents in 114 (37.5%) (Table 3).

### 3.3. LTBI Prevalence and Associated Factors

All 304 patients underwent QFN-Plus testing, and 230 (75.6%) had a TST test. The LTBI prevalence according to QFT-Plus was 34.2% (95% CI 29.1–39.7%, n = 104). Among the 230 patients with TST results, 82 had a positive result (induration ≥5 mm), yielding a prevalence of 35.6% (95% CI 29.7–42.0%). Bivariate analysis identified prior TB exposure (OR 4.19, 95% CI 1.79–9.78, *p* < 0.001); prior anti-TNF-α use (OR 1.40, 95% CI 1.00–1.97, *p* = 0.038) associated with LTBI; and RA (OR 0.38, 95% CI 0.022–0.65, *p* < 0.001) as a lower frequency of positive QFT Gold Plus. In multivariate analysis, prior TB exposure (aOR 4.20, 95% CI 1.74–10.12, *p* = 0.001) was a significant risk factor for LTBI, while rheumatoid arthritis was associated with a lower likelihood of having positive QFT-Plus (aOR 0.31, 95% CI 0.16–0.59, *p* < 0.001). No significant associations were found for age, sex, educational level, comorbidities, tobacco or alcohol use, BCG vaccination, or other DMARDs (Table 4 and Table 5).

### 3.4. Concordance Between QTF Plus and TST

The kappa coefficient between QFT-plus and the TST was 0.52, which shows a moderate grade of agreement.

## 4. Discussion

This cross-sectional study conducted at the Hospital de Infectología, National Medical Center “La Raza,” in Mexico City, provides critical insights into the prevalence of LTBI among patients with IMIDs in a high-TB-burden setting. The observed LTBI prevalence using QFT-Plus and TST underscores a significant burden of LTBI in this population, particularly among those with prior TB exposure.

These findings align with regional data from Latin America, where high TB incidence and widespread BCG vaccination complicate LTBI diagnosis and management. The study’s results emphasize the importance of targeted screening and preventive strategies for IMID patients, especially for those initiating or using DMARDs, which are known to increase the risk of TB reactivation.

The observed LTBI prevalence exceeds the global estimate of 24.8% obtained using IGRAs and Mexico’s national estimate of 19–20% [7]. This discrepancy may reflect the inclusion of diverse IMIDs, with dermatological conditions like psoriasis (41.4%) predominating, compared to prior studies focused only on RA [16].

Compared to Latin American studies, our study reports a higher prevalence than the 29.5% in Brazil and 29% in Peru, both of which were obtained using the TST [11,12]. We used QFT-Plus and TST for testing. The elevated prevalence observed with TSTs may be linked to false positives associated with widespread BCG vaccination in several Latin American countries, as well as the inclusion of patients with comorbidities such as diabetes and hypertension, which were excluded in previous studies [8,9,10]. Additionally, in this study, the majority of patients with immune-mediated inflammatory diseases (IMIDs) were those with dermatological conditions, mainly psoriasis. This likely contributed to the higher prevalence, highlighting an underrepresented population in global latent tuberculosis infection (LTBI) estimates.

Multivariate analysis identified prior TB exposure as the strongest independent risk factor for LTBI (aOR 4.20, 95% CI 1.74–10.12, *p* = 0.001), consistent with established literature linking close contact with active TB cases to increased infection risk [23]. This finding is particularly relevant in Mexico, where urban areas like Mexico City and high-incidence states (e.g., Veracruz, Guerrero) facilitate TB transmission due to overcrowding and socioeconomic challenges [17]. The lack of association with other factors, such as age, sex, or comorbidities like diabetes, contrasts with some studies that report diabetes as a risk factor for LTBI [24]. This may be due to the specific immunosuppressive profile of IMID patients, which may overshadow metabolic risk factors in this study.

Interestingly, rheumatoid arthritis was associated with a lower likelihood of positive QFT-Plus results. This unexpected finding may reflect disease-specific immune dysregulation or differences in treatment regimens, as RA patients in our study were less likely to have received anti-TNF-α agents compared to those with psoriasis (59.5% overall, with adalimumab predominant).

Notably, 90.1% of patients resided in central Mexico, a region with lower TB incidence (≤15.7 cases/100,000 inhabitants) compared to national averages. Yet, the LTBI prevalence matched that of high-burden regions like border areas, where sociodemographic factors such as occupational exposure, overcrowding, and proximity to migrant populations elevate risk [18,25]. This suggests that IMID patients, particularly those on immunosuppressive therapies, face heightened LTBI risk regardless of regional TB incidence, possibly due to hospital-based referral patterns in our study.

In this study, prior use of anti-TNF-α agents was associated with LTBI positivity in bivariate analysis (OR 1.40, 95% CI 1.00–1.97, *p* = 0.038) but did not retain significance in multivariate analysis (aOR 0.70, 95% CI 0.41–1.20, *p* = 0.20). While anti-TNF-α therapies, such as adalimumab and infliximab, are well-established risk factors for the reactivation of LTBI to active tuberculosis due to suppressing TNF-α, a cytokine critical for maintaining granuloma integrity and controlling *Mycobacterium tuberculosis*, TNF-α facilitates immune cell recruitment and granuloma formation, which contain latent TB bacteria. By inhibiting TNF-α, these drugs disrupt granulomas, potentially releasing dormant bacteria and leading to reactivation [26]. Their association with an increased likelihood of LTBI positivity on tests like QFT-Plus is less direct, as these agents are not expected to increase the likelihood of acquiring a latent infection. The association observed in bivariate analysis may be explained by indirect factors. For instance, patients receiving anti-TNF-α therapy may have had greater prior exposure to *Mycobacterium tuberculosis*, a dominant risk factor for LTBI, potentially due to a more severe IMID or referral to our center for study, increasing the likelihood of a positive QFT-Plus result. Additionally, the timing of LTBI screening may have influenced findings, as 54.9% of patients were tested before initiating biologic DMARDs, potentially limiting the detection of anti-TNF-α’s immunosuppressive effects on QFT-Plus performance or LTBI reactivation risk. Furthermore, heterogeneity in anti-TNF-α agents (e.g., adalimumab in 43.4% of cases) and variability in treatment duration may have diluted the association, as different agents and exposure durations could exert varying immunosuppressive effects [27]. Finally, the sample size (n = 304) may have limited the statistical power to detect a significant association in the multivariate model, particularly when adjusting for covariates like prior TB exposure. These findings suggest that while anti-TNF-α therapy does not directly increase the risk of acquiring LTBI, its association with positive QFT-Plus results in this study likely reflects confounding factors, such as TB exposure, rather than a direct effect on latent infection. Longitudinal studies with detailed data on treatment duration and TB exposure timing are needed to further clarify the relationship between anti-TNF-α use and LTBI prevalence in IMID populations in high-TB-burden settings. Given the associated risks, anti-TNF-α agents are sometimes avoided in patients with IMIDs and LTBIs.

Immunosuppressants with safer TB reactivation profiles, such as anti-IL-17 (secukinumab) and anti-IL-23 agents [28,29], were less frequently prescribed in this study.

The finding that 45.1% of patients had prior DMARD use, with 37.5% having received anti-TNF-α agents, highlights the chronic nature of IMIDs and the cumulative immunosuppressive burden in this population. This is particularly concerning in high-TB-burden settings, where prolonged immunosuppression increases reactivation risk [30]. The absence of significant associations with other DMARDs, such as rituximab or IL-17 inhibitors, may reflect their lower impact on TB immunity compared to TNF-α inhibitors, as supported by prior studies [31].

Several factors have been associated with indeterminate or negative IGRA results in immunocompromised populations. In this study, no association was observed between negative QFT-Plus results and corticosteroid use at doses ≥10 mg/day of prednisone or an equivalent, contrasting with findings by Bélard et al., who reported a 27% increased risk of indeterminate IGRA results with 10 mg/day of prednisolone [32]. This may reflect differences in patient populations, immunosuppressive regimens, or the specific IGRA used, as Bélard et al. employed the QuantiFERON Gold In-Tube (QFT-GIT) assay, which may have lower sensitivity in immunocompromised individuals.

Age is another factor potentially influencing IGRA results. Tebruegge et al. noted a negative correlation between interferon-gamma concentrations and advanced age, with higher rates of indeterminate results in elderly populations according to QFT-GIT results [33]. Similarly, Jung-Yien-Chien et al. reported decreased QFT-GIT sensitivity with increasing age [34]. However, in our study, no association was found between negative QFT-Plus results and older age (>50 years), consistent with reports indicating that QFT-Plus maintains high sensitivity (100% overall, 94% in those ≥75 years) across age groups [34]. This suggests that QFT-Plus may perform more reliably in older IMID patients compared to QFT-GIT.

The type of IMID may also affect IGRA outcomes. QFT-Plus has demonstrated improved sensitivity in IMID patients compared to QFT-GIT, with indeterminate result rates of 0.7% versus 5.2%, respectively, as reported by Igari et al., potentially due to lower CD4+ and CD8+ lymphocyte counts in immunocompromised individuals [35]. Although CD4+ and CD8+ counts were not measured in our study, an inverse association was observed between RA and positive QFT-Plus results. This finding may reflect RA-specific immunological factors or differences in treatment regimens, warranting further investigation into disease-specific effects on IGRA performance.

Prior TB exposure is a well-documented risk factor, with household contacts at elevated risk [36,37]. The strong association with TST ≥5 mm supports its utility as a screening tool, despite its reduced specificity in BCG-vaccinated populations or those on glucocorticoids or synthetic DMARDs [38]. The high concordance between the TST and IGRA in this cross-sectional study suggests that the TST remains valuable when the IGRA is unavailable, though the IGRA’s superior performance is preferred [8,38].

A study on LTBI prevalence in patients with IMIDs reported a prevalence of 31.7%, with a concordance between the TST and IGRA of 0.62 (kappa). Similarly, the TITAN study found an LTBI prevalence of approximately 35%, with a concordance between QFT-Plus and the TST of 0.52 (kappa). The moderate concordance (kappa ≈ 0.5–0.6) in both cross-sectional studies suggests discrepancies that are likely due to factors such as prior BCG vaccination or non-tuberculous mycobacterial infections. Variations in prevalence and concordance may also stem from differences in the IMID populations analyzed, highlighting that test selection can influence LTBI detection depending on the population context [39].

This study has some limitations. First, the cross-sectional design limits the exploration of other factors for LTBI. Second, recall bias may affect self-reported TB exposure data. Third, in some cases, patients were already receiving biologic DMARDs, so we cannot exclude the impact of these drugs from the performance of diagnostic tests. Immunosuppression with prednisone could impact the results of the TST and IGRA. Finally, the hospital-based sample, predominantly from central Mexico, may limit generalizability to other regions.

The strength of this study was the use of QFT-TB gold Plus to enhance diagnostic accuracy, addressing a gap in Latin American studies, where the IGRA is rarely used. Comprehensive data on BCG vaccination, comorbidities, and sociodemographic factors (e.g., occupation, residence) provide a robust context for LTBI risk, distinguishing this study from prior work on immunocompromised populations.

Future research should focus on longitudinal studies to assess the incidence of TB reactivation in IMID patients with LTBI, particularly those on anti-TNF-α therapy. Comparative studies across Latin American countries could elucidate regional differences in LTBI prevalence and risk factors, informing region-specific guidelines. Additionally, cost-effectiveness analyses of the IGRA versus TST in resource-limited settings could guide policy decisions to improve diagnostic access. Investigating the immunological mechanisms underlying the lower LTBI prevalence in RA patients could provide insights into disease-specific risk profiles and optimize screening strategies.

## 5. Conclusions

This study identified a high prevalence of latent tuberculosis infection (LTBI) among patients with immune-mediated inflammatory diseases (IMIDs) globally, with 34.2% (95% CI 29.1–39.7%) testing positive via QuantiFERON-TB Gold Plus and 35.6% (95% CI 29.7–42.0%) via TSTs. Prior TB exposure emerged as the strongest independent risk factor for LTBI (aOR 4.20, 95% CI 1.74–10.12, *p* = 0.001), underscoring the critical influence of environmental and contact history in TB-endemic regions worldwide. Patients with rheumatoid arthritis (RA) showed a lower likelihood of positive QuantiFERON-TB Gold Plus results. These findings highlight the global importance of routine LTBI screening using sensitive diagnostic tools like the IGRA in IMID populations, particularly for those on biologic therapies, to inform targeted TB prevention strategies and reduce the risk of reactivation across diverse high-risk patients.

## Figures and Tables

**Table 1:** Clinical and sociodemographic characteristics (N = 304).

Characteristic	Value
Age, years, median (IQR)	53 (39–61)
Sex, male, n (%)	154 (50.7)
Education level, n (%)	
Unlearned	1 (0.3)
Primary school	23 (7.5)
High school	69 (22.7)
Preparatory school/technical career	109 (35.8)
University degree	88 (28.9)
Postgraduate	14 (4.6)
Occupation, n (%) ^(a)^	
Merchants	84 (27.6)
Professionals	60 (19.7)
Domestic services	35 (11.5)
Administrative services	34 (11.1)
Industrial or technical services	24 (7.8)
Students	21 (6.9)
Region of origin/birth, n (%) ^(a)^	
Mexico City	196 (64.4)
Mexico State	53 (17.4)
Veracruz	11 (3.6)
Michoacan	8 (2.6)
Oaxaca	6 (2.0)
Puebla	5 (1.6)
Tlaxcala	4 (1.3)
Guerrero	4 (1.3)
Guanajuato	3 (1.0)
San Luis Potosi	2 (0.7)
Nuevo León	2 (0.7)
Another country	2 (0.7)
Comorbidities, n (%) ^(a)^	
Systemic arterial hypertension	94 (30.9)
Type 2 diabetes	68 (22.4)
Metabolic syndrome	25 (8.2)
Hypothyroidism	15 (4.9)
Heart diseases ^(b)^	10 (3.3)
Chronic kidney disease	8 (2.6)
Asthma	5 (1.6)
Chronic obstructive pulmonary disease	4 (1.3)
Tobacco use, n (%)	104 (34.5)
Alcohol consumption, n (%)	96 (31.6)
Infections, n (%)	
HIV	3 (1.0)
HCV	1 (0.3)
HBV	1 (0.3)
TST ^(c)^	
TST test, regardless of the result	230 (75.6%)
TST > 5 mm	82 (35.6%)

^(a)^ The most frequently encountered characteristics were described. ^(b)^ Ischemic heart disease and heart failure were included. ^(c)^ Tuberculin skin test.

**Table 2:** Distribution of cases of immune-mediated diseases (N = 304).

	LTBI (n = 104)	No LTBI (n = 200)
IMID	**n (%)**	**n (%)**
Psoriasis	38 (36.5)	88 (44.0)
Rheumatoid arthritis	27 (26.0)	20 (10.0)
Ankylosing spondylitis	13 (12.5)	18 (9.0)
Psoriatic arthritis	7 (6.7)	13 (6.5)
Hidradenitis suppurativa	4 (3.8)	27 (13.5)
Pemphigus	4 (3.8)	6 (3.0)
Atopic dermatitis	3 (2.9)	16 (8.0)
Inflammatory bowel disease	3 (2.9)	7 (3.5)
Multiple sclerosis	3 (2.9)	0 (0.0)
Systemic lupus erythematosus	1 (1.0)	2 (1.0)
Others ^(a)^	1 (1.0)	3 (1.5)

^(a)^ Still’s disease, Polymyalgia rheumatica, Systemic sclerosis.

**Table 3:** Distribution of prescription/use of biologic DMARDs (N = 304).

DMARDs to Be Started, n (%) ^(a)^	n (%)	Previously Used DMARDs, n (%) ^(b)^	n (%)
Adalimumab	132 (43.4)	Adalimumab	91 (29.9)
Secukinumab	46 (15.1)	Secukinumab	9 (3.0)
Baricitinib	21 (6.9)	Infliximab	7 (2.3)
Rituximab	19 (6.2)	Rituximab	7 (2.3)
Golimumab	19 (6.2)	Certolizumab	5 (1.6)
Infliximab	12 (3.9)	Golimumab	5 (1.6)
Certolizumab	12 (3.9)	Etanercept	4 (1.3)
Ustekinumab	11 (3.6)	Abatacept	2 (0.7)
Ixekinumab	7 (2.3)	Interferon 1 Beta	2 (0.7)
Etarnercept	4 (1.3)	Ixekizumab	2 (0.7)
Abatacept	3 (0.9)	Tocilizumab	2 (0.7)
Tocilizumab	2 (0.6)	Glatiramer acetate	1 (0.3)
Cladribina	2 (0.6)	Baricitinib	1 (0.3)
Guselkumab	1 (0.3)	Fingolimod	1 (0.3)
Tofacitinib	1 (0.3)		

^(a)^ The DMARD to be used was not specified in 12 cases. ^(b)^ 165 cases had no history of prior biological FARME use.

**Table 4:** Bivariate analysis of possible factors associated with LTBI.

Associated Factors	OR	*p* Value
Sex (male)	1.24 (0.77–1.99)	0.37
Age (>50 years)	1.03 (0.64–1.66)	0.89
Age (<50 years)	1.04 (0.70–1.30)	0.77
Basic education level	0.82 (0.48–1.38)	0.46
Systemic arterial hypertension	1.13 (0.68–1.88)	0.63
Type 2 diabetes	1.47 (0.84–2.56)	0.16
Metabolic syndrome	1.31 (0.56–3.03)	0.52
Hypothyroidism	0.96 (0.31–2.88)	0.94
Heart diseases	1.29 (0.35–4.68)	0.69
Chronic obstructive pulmonary disease	0.63 (0.06–6.20)	0.69
Chronic kidney disease	0.26 (0.03–2.20)	0.19
Tobacco use	1.00 (0.61–1.65)	0.98
Alcohol consumption	1.32 (0.79–2.18)	0.28
HIV infection	1.94 (0.27–13.98)	0.50
HCV infection	3.94 (0.35–43.97)	0.23
Born in a high-TB incidence region	1.36 (0.56–3.30)	0.49
BCG vaccination history	0.91 (0.39–2.15)	0.84
Rheumatoid arthritis	0.385 (0.227–0.653)	<0.001
Prior TB exposure	4.19 (1.79–9.78)	<0.001
Prior synthetic DMARD use	1.08 (0.66–1.77)	0.74
Prior biologic DMARD use	0.91 (0.56–s1.46)	0.70
Anti-TNF-α use	1.40 (1.00–1.97)	0.038
Rituximab	2.62 (0.57–11.96)	0.19
IL-17 inhibitors	1.63 (0.48–5.48)	0.42
Prednisone ≥ 10 mg/day	0.982 (0.584–1.65)	0.94

**Table 5:** Multivariate analysis of possible factors associated with LTBI.

Associated Factors	aOR	*p* Value
Type 2 diabetes	1.69 (0.94–3.03)	0.07
Chronic kidney disease	0.27 (0.03–2.38)	0.23
HCV infection	4.46 (0.38–51.50)	0.23
Rheumatoid arthritis	0.317 (0.168–0.599)	<0.001
Prior TB exposure	4.20 (1.74–10.12)	0.001
Anti-TNF-α use	0.70 (0.41–1.20)	0.20
Rituximab	2.33 (0.42–13.01)	0.33

## Data Availability

Data are unavailable due to privacy or ethical restrictions. Data are available upon reasonable request from the corresponding author.

## Abbreviations

The following abbreviations are used in this manuscript:

Anti-TNF-α	Tumor necrosis factor-alpha inhibitors
aOR	Adjusted odds ratio
BCG	Bacillus Calmette-Guérin
CI	Confidence interval
DMARDs	Disease-modifying antirheumatic drugs
HBV	Hepatitis B virus
HCV	Hepatitis C virus
HIV	Human immunodeficiency virus
IGRAs	Interferon-gamma release assays
IMIDs	Immune-mediated inflammatory diseases
JAKi	Janus kinase inhibitors
LTBI	Latent tuberculosis infection
OR	Odds ratio
QFT-Plus	QuantiFERON-TB Gold Plus
TB	Tuberculosis
TNF-α	Tumor necrosis factor-alpha
TST	Tuberculin skin test
